# Niveles de ferritina en artritis idiopática juvenil de inicio sistémico comparada con otras fiebres de origen desconocido: estudio multicéntrico de pruebas diagnósticas

**DOI:** 10.7705/biomedica.5849

**Published:** 2021-12-15

**Authors:** Ruth Eraso, Claudia Patricia Benitez, Sergio Jaramillo, Jorge Acosta-Reyes, Beatriz Helena Aristizábal, Augusto Quevedo

**Affiliations:** 1 Facultad de Medicina, Departamento de Pediatría, Universidad de Antioquia, Medellin, Colombia Universidad de Antioquia Facultad de Medicina Departamento de Pediatría Universidad de Antioquia Medellin Colombia; 2 Departamento de Pediatría, Hospital Pablo Tobón Uribe, Medellin, Colombia Departamento de Pediatría Hospital Pablo Tobón Uribe Medellin Colombia; 3 Unidad de Investigación Genética Molecular, UNIGEM-Colombia, Medellin, Colombia Unidad de Investigación Genética Molecular UNIGEM-Colombia Medellin Colombia; 4 Departamento de Laboratorio, Hospital Pablo Tobón Uribe, Medellin, Colombia Departamento de Laboratorio Hospital Pablo Tobón Uribe Colombia; 5 División Ciencias de la Salud, Departamento de Salud Pública, Universidad del Norte, Barranquilla, Colombia Universidad del Norte División Ciencias de la Salud Departamento de Salud Pública Universidad del Norte Barranquilla Colombia; 6 Departamento de Pediatría, Hospital Universitario San Vicente Fundación, Medellin, Colombia Departamento de Pediatría Hospital Universitario San Vicente Fundación Medellin Colombia

**Keywords:** artritis idiopática juvenil sistémica/diagnóstico, ferritinas, sensibilidad y especificidad, cociente de probabilidades, systemic juvenile idiopathic arthritis/diagnosis, ferritins, sensitivity and specificity, likelihood ratio

## Abstract

**Introducción.:**

No se dispone de pruebas sensibles ni específicas para diagnosticar la artritis idiopática juvenil sistémica.

**Objetivo.:**

Evaluar la utilidad diagnóstica de niveles de ferritina total cinco veces por encima del valor normal (ferritina total>5N) y el porcentaje disminuido (menor de o igual a 20 % de la ferritina total) de la ferritina glucosilada (ferritina glucosilada<20 %) para el diagnóstico de artritis idiopática juvenil sistémica en pacientes con fiebre de origen desconocido evaluados por Reumatología pediátrica.

**Materiales y métodos.:**

Se hizo un estudio observacional de pruebas diagnósticas de corte transversal en menores de 16 años hospitalizados entre el 2010 y el 2014. El patrón diagnóstico de referencia fue el cumplimiento de los criterios de clasificación o diagnóstico confirmado en el seguimiento. Se determinaron las medidas de utilidad de las pruebas.

**Resultados.:**

Se incluyeron 40 pacientes con fiebre de origen desconocido: 11 con artritis idiopática juvenil sistémica y 29 con otros diagnósticos. La mediana de la ferritina total fue mayor en la artritis idiopática juvenil sistémica (3.992 ng/ml) comparada con otras causas de fiebre de origen desconocido (155 ng/ml) (p=0,0027), así como la ferritina total>5N (90,91 % Vs. 51,72 %) (p=0,023). El porcentaje de ferritina glucosilada≤20 % fue de 96,5 % en otras fiebres de origen desconocido en comparación con la artritis idiopática juvenil sistémica (81,8 %) (p=0,178). La ferritina total>5N tuvo una sensibilidad del 91 %, una especificidad del 48 %; un cociente de probabilidades (*Likelihood Ratio*, LR) positivo de 1,76 y uno negativo de 0,19, demostrando mayor utilidad para el diagnóstico que la combinación de la ferritina total>5N y ferritina glucosilada<20 %, cuya sensibilidad fue del 81,8 %, la especificidad del 48,3 %, un cociente de probabilidades LR positivo de 1,58 y un LR negativo de 0,38.

**Conclusión.:**

En pacientes con fiebre de origen desconocido evaluados por reumatología pediátrica, la ferritina total>5N demostró ser útil como prueba de tamización para el diagnóstico de artritis idiopática juvenil sistémica.

## Abstract (en)

**Introduction:**

There are no sensitive or specific tests available to diagnose systemic juvenile idiopathic arthritis (sJIA).

**Objective:**

To assess the utility as diagnostic tests of total ferritin (TF) levels greater than 5 times the normal value (TF>5N) and the decreased percentage (less than or equal to 20% of TF) of glycosylated ferritin (GF≤20%) for the diagnosis of sJIA in patients with fever of unknown origin evaluated by pediatric rheumatology.

**Materials and methods:**

We conducted an observational, cross-sectional study of diagnostic tests in children under 16 years of age hospitalized between 2010 and 2014. The reference diagnostic standard was the fulfillment of the classification criteria or confirmed diagnosis at follow-up. We determined the measures of utility of the tests.

**Results:**

We included 40 patients with fever of unknown origin, 11 with sJIA, and 29 with other diagnoses. The median TF was higher in sAIJ (3992 ng/ml) versus other causes of fever of unknown origin (155 ng/ml) (p=0.0027), as well asTF>5N (90.91% versus 51.72%) (p=0.023). The percentage of GF≤20% was higher in patients with other causes of fever of unknown origin (96.5%) compared to sJIA (81.8%) (p=0.178).TF>5N had a sensitivity of 91%, specificity of 48%, positive likelihood ratio (LR) of 1.76, and negative LR of 0.19 demonstrating greater utility for the diagnosis of sJIA than the combination of FT> 5N with GFR <20%, with a sensitivity of 81.8%, specificity of 48.3%, positive LR of 1.58, and negative LR of 0.38.

**Conclusion:**

In patients with FUO evaluated by pediatric rheumatology, TF> 5N proved useful as a screening test for the diagnosis of sJIA.

La artritis idiopática juvenil sistémica es una enfermedad autoinflamatoria de etiología desconocida que, además de la artritis, se caracteriza por fiebre en picos, exantema maculopapular evanescente, linfadenopatías, hepatoesplenomegalia y serositis ^1^. La presentación clínica es diversa; algunos pacientes no presentan artritis hasta semanas o meses después del inicio de la fiebre, otros manifiestan patrones de fiebre atípicos o no tienen ninguna otra manifestación sistémica además de la fiebre. Aproximadamente el 10 % de los pacientes presenta como complicación una forma de linfohistiocitosis hemofagocítica secundaria, denominada síndrome de activación macrofágica, que puede ser fatal ^2^. En la mayoría de los casos, los resultados de los exámenes de laboratorio, incluido el aumento de la velocidad de eritrosedimentación, de la proteína C reactiva, la leucocitosis, la neutrofilia y la trombocitosis, reflejan el estado inflamatorio; sin embargo, no se dispone de pruebas sensibles ni específicas de apoyo para el diagnóstico de la artritis idiopática juvenil sistémica. Dada la naturaleza inespecífica de los signos y síntomas y de las pruebas de laboratorio, el enfoque clínico es difícil y el diagnóstico se hace por exclusión, lo que implica descartar una gran cantidad de enfermedades que comparten manifestaciones e incluyen todas aquellas que se manifiestan como fiebre de origen desconocido.

La *International League of Associations for Rheumatology* (ILAR) estableció unos criterios de clasificación para la artritis idiopática juvenil sistémica ^3^, sin embargo, estos establecen como condición que el paciente presente artritis y que se descarten otros diagnósticos, por lo que son poco útiles en la práctica clínica y han sido objeto de cuestionamientos ^4^. En una cohorte de 136 pacientes con artritis idiopática juvenil sistémica, solo 42 de 136 (30 %) cumplieron dichos criterios ^5^. Los datos provenientes de dos registros alemanes indicaron que estos criterios solo detectaron el 47,8 y el 54,3 % de los casos, respectivamente ^6^. Recientemente, el grupo de la *Pediatric Rheumatology International Trials Organization* (PRINTO) propuso una nueva clasificación de la artritis idiopática juvenil que incluye criterios para la forma sistémica, pero no contempla la artritis como un criterio obligatorio; no obstante, esta es una propuesta que aún no ha sido validada ni aceptada de forma universal ^7^.

Para el enfoque de los pacientes con fiebre de origen desconocido, se han propuesto diferentes marcadores biológicos con el fin de estrechar el espectro de posibilidades diagnósticas, entre los que se cuentan la proteína C reactiva y la procalcitonina para predecir infecciones bacterianas ^8^, las sustancias producidas por macrófagos activados como las proteínas S100A12 y S100A8/ A9 (calprotectina) para la artritis idiopática juvenil sistémica ^9^ y la ferritina total y la glucosilada para la enfermedad de Still del adulto ^10^.

La ferritina es la principal forma de depósito de hierro intracelular, pero sus niveles no solo se correlacionan con el hierro almacenado, sino también, con estados de enfermedad como reactante de fase aguda ^11^. Aunque la mayor Ferritina en artritis idiopática juvenil y en otras fiebres de origen desconocido parte de la ferritina está dentro de la célula, existe una cantidad significativa en el plasma; una porción de esta ferritina circulante puede provenir del escape de células necróticas, pero la mayoría se produce por secreción, proceso durante el cual se le adicionan moléculas de carbohidratos ^12^. Por esta razón, en condiciones normales la ferritina circulante está glucosilada en 45 a 70 %, en tanto que la ferritina intracelular no tiene carbohidratos ^13^. Esta relación se altera en estados inflamatorios y hepatopatías, en los cuales puede elevarse desproporcionadamente la ferritina circulante ^14^. Sin embargo, en estas condiciones, aunque los niveles de ferritina total se aumentan, el porcentaje de ferritina glucosilada con frecuencia permanece bajo y no reacciona con el mismo estímulo ^15^.

Como la ferritina aumenta en la mayoría de estados inflamatorios, se considera muy sensible para detectar la inflamación, pero muy poco específica para diferenciar entre las numerosas causas que la explican ^16^^-^^18^. Sin embargo, llama la atención su notorio aumento en la enfermedad de Still del adulto, considerada por la mayoría de los autores como la enfermedad equivalente a la artritis idiopática juvenil sistémica en pacientes mayores de 16 años ^19^. En dicha enfermedad, la ferritina total generalmente aumenta por encima de cinco veces su valor normal, dato que ayuda al diagnóstico diferencial en el contexto clínico apropiado. Varios estudios, incluido uno en Colombia ^20^, demuestran la utilidad diagnóstica tanto del aumento de la ferritina total como de la disminución muy marcada en el porcentaje de la ferritina glucosilada ^10^^,^^20^^-^^23^. Además, estos marcadores son útiles en el diagnóstico de las formas primarias y secundarias de linfohistiocitosis hemofagocítica ^24^.

Fautrel, *et al.*, encontraron que la combinación de la elevación de la ferritina total 5 veces por encima del valor normal y un valor de ferritina glucosilada menor o igual al 20 % de la ferritina total tiene una sensibilidad del 43,2 % y una especificidad del 92,9 % para detectar la enfermedad de Still del adulto ^10^. En el 2002 se incluyó la ferritina glucosilada menor de o igual al 20 % de la ferritina total como uno de los principales criterios de una propuesta de clasificación de esta enfermedad ^25^, los cuales fueron validados en el 2018 ^26^. Los estudios mencionados se hicieron en pacientes con enfermedad de Still del adulto, en tanto que la información sobre la ferritina en la artritis idiopática juvenil sistémica es escasa y se limita a estudios descriptivos ^5^^,^^27^^,^^28^.

Después de una búsqueda exhaustiva, no se encontraron estudios en niños que compararan los niveles de ferritina en la artritis idiopática juvenil sistémica con los observados en otras fiebres de origen desconocido, como tampoco estudios que evaluaran la utilidad de estas pruebas en el diagnóstico de la artritis idiopática juvenil sistémica.

En este contexto, el objetivo de este estudio fue evaluar la utilidad y el rendimiento de los niveles de ferritina total cinco veces por encima del valor normal (según el grupo de edad y el sexo) y el porcentaje disminuido (menor de o igual al 20% de la total) de la ferritina glucosilada como pruebas para el diagnóstico de la artritis idiopática juvenil sistémica en pacientes con fiebre de origen desconocido evaluados por reumatología pediátrica en dos centros de referencia de Medellín, Colombia.

## Materiales y métodos

Se hizo un estudio de pruebas diagnósticas de tipo observacional y corte transversal en dos instituciones de cuarto nivel de atención, las cuales son centros de referencia de reumatología pediátrica en Medellín, Colombia: el Hospital Pablo Tobón Uribe y el Hospital Universitario San Vicente Fundación. Se incluyeron de forma prospectiva pacientes menores de 16 años hospitalizados entre el 1^o^ de noviembre de 2010 y el 1^o^ de febrero de 2014 con solicitud de interconsulta en reumatología pediátrica por la presencia de fiebre. Se consideraron como pacientes con fiebre de origen desconocido a aquellos con temperatura axilar mayor de 38 °C, por lo menos, dos veces a la semana durante más de dos semanas a quienes no se había podido diagnosticar después de la evaluación clínica exhaustiva y de haber realizado las siguientes pruebas: hemograma, velocidad de eritrosedimentación, proteína C reactiva, citoquímico de orina, urocultivo, hemocultivos, y rayos X de tórax ^29^^,^^30^. La velocidad de eritrosedimentación se determinó mediante técnica automatizada por fotometría capilar, con valores de referencia de 1 a 20 mm/hora, y la proteína C reactiva por inmunoturbidimetría con valores de referencia de 0,01 a 0,82 mg/dl. Se excluyeron los pacientes con antecedentes de ingestión de hierro farmacológico, deferoxamina o transfusión en los últimos seis meses, enfermedad renal crónica, hepatopatía grave o inmunodeficiencia primaria confirmada.

A cada paciente se le hizo una evaluación clínica completa con énfasis en las características de la fiebre y se ordenaron los exámenes pertinentes para el manejo de la fiebre de origen desconocido, incluida la medición de la ferritina total y la glucosilada según la metodología que se describe más adelante. Durante la hospitalización, se hizo seguimiento de los pacientes y, en el momento del alta, se dejó consignado si había sido posible determinar el diagnóstico definitivo de la fiebre. A los pacientes con diagnóstico de probable artritis idiopática juvenil sistémica en el momento del alta, se les hizo seguimiento clínico, por lo menos, durante 12 meses, con evaluación clínica periódica por reumatología pediátrica hasta confirmar o descartar el diagnóstico. A los pacientes sin ningún diagnóstico definido o con diagnósticos diferentes a la artritis idiopática juvenil sistémica en el momento del alta, se les hizo seguimiento telefónico de seis a 12 meses a partir del egreso hospitalario para determinar el diagnóstico definitivo de la causa de la fiebre.

El diagnóstico definitivo de artritis idiopática juvenil sistémica se consideró de acuerdo con los siguientes parámetros:

Pacientes que cumplían los siguientes criterios de la ILAR ^3^: artritis en una o más articulaciones, con (o precedida por) fiebre diaria mayor de o igual a 39 °C de, por lo menos, dos semanas de duración, cotidiana por lo menos durante tres días y acompañada de uno o más de los siguientes signos: exantema típico, linfadenopatías, hepatoesplenomegalia o serositis; se excluyeron los pacientes positivos para el factor reumatoide, por lo menos, en dos ocasiones y con antecedente personal o familiar en primer grado de consanguinidad de enfermedades asociadas con el antígeno HLA-B27 (espondilitis anquilosante, artritis asociada a entesitis, artritis reactiva, sacroiliítis con enfermedad inflamatoria intestinal, uveítis anterior aguda, psoriasis o artritis psoriásica).

Pacientes que, sin cumplir los anteriores criterios, después de al menos 12 meses de seguimiento por reumatología pediátrica, continuaban dependiendo de tratamiento con inmunomoduladores para el control de los síntomas y sin otro diagnóstico que explicara su cuadro clínico.

Para el análisis de los datos, se excluyeron aquellos pacientes sin diagnóstico definido al darlos de alta y sin seguimiento ambulatorio ni posibilidad de contactarlos en el momento de la búsqueda para determinar el diagnóstico definitivo a los seis meses de su inclusión en el estudio. En la fase de selección de pacientes para el estudio, el pediatra reumatólogo no tuvo acceso a los resultados de la ferritina glucosilada.

Como la muestra de la que se partió para este estudio no era población pediátrica general sino pacientes con diagnóstico establecido de fiebre de origen desconocido, se esperaba que el mayor valor de la prueba ayudara a confirmar la enfermedad, es decir, que tuviera una gran especificidad. Por ello, para el cálculo del tamaño de la muestra se planteó una sensibilidad del 50 % y una especificidad del 95 %. Se utilizó la prevalencia reportada de artritis idiopática juvenil sistémica del 5,5 % en pacientes pediátricos con fiebre de origen desconocido en el estudio de revisión sistemática de Chow, *et al.* (31). Se calculó un error estándar del intervalo de confianza del 2,1 a 2,5 % (precisión). El tamaño de muestra obtenido con el programa Epidat 3,1 fue entre 27 y 36 pacientes, y se tomó el dato más preciso de 36 pacientes. El cálculo del tamaño de muestra se hizo planteando una sensibilidad del 90 % y una especificidad del 50 %, obteniéndose un tamaño muestral de 37 pacientes. La información de cada paciente se recogió en un formato diseñado para el estudio. Todos los datos se ingresaron en una hoja de cálculo de Excel© y se hizo el análisis estadístico con el paquete MedCalc Statistical Software, versión 19.4.0.

### Determinación de la ferritina sérica total y el porcentaje glucosilado

La ferritina sérica total es la concentración en plasma expresada en ng/ mi medida por inmunoensayo; la ferritina glucosilada es la forma de ferritina asociada con carbohidratos que constituye normalmente entre el 45 y el 70 % de la ferritina circulante. La medición de la ferritina glucosilada se hizo con la técnica de Worwood ^32^ (anexo 1).

A los valores obtenidos se les aplicó la siguiente fórmula para determinar el porcentaje de ferritina glucosilada:

**Figure:**
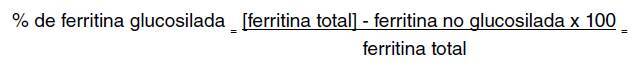

Los niveles de ferritina total se consideraron elevados cuando se encontraban por encima de los estándares normales según la edad, así: para niños de ambos sexos de 1 a 5 años, hasta 24 ng/ml, y de 6 a 10 años, hasta 55 ng/ml; de los 11 a los 16 años, para el sexo masculino hasta 70 ng/ml y para el femenino hasta 40 ng/ml ^33^.

#### Análisis estadístico

Se utilizó como patrón diagnóstico de referencia de la artritis idiopática juvenil sistémica el cumplimiento de los criterios de la ILAR o el diagnóstico confirmado con base en la evolución del paciente durante un seguimiento, por lo menos, de 12 meses a cargo del especialista en reumatología pediátrica y con base en los criterios anotados.

Se determinó la distribución normal de las variables mediante la prueba de Shapiro-Wilks. Las variables con distribución normal se expresaron con medias y desviación estándar; las variables que no cumplieron este supuesto se expresaron como medianas y rangos intercuartiles (RIC) y, las variables categóricas, en números absolutos y relativos. Se compararon las frecuencias entre los grupos con artritis idiopática juvenil sistémica y otras causas de fiebre de origen desconocido con la prueba de ji al cuadrado o la exacta de Fisher según los valores esperados. Los promedios se compararon con una prueba U de Mann-Whitney. Los valores de p<0,05 se consideraron estadísticamente significativos.

Se hicieron tablas de contingencia con los siguientes valores: ferritina total cinco veces por encima del límite superior esperado para la edad y el sexo; ferritina glucosilada menor de o igual al 20 % de la ferritina total, y la combinación de ambas mediciones. Se calcularon los valores de sensibilidad, especificidad, los valores predictivos, la exactitud y los cocientes de probabilidades (LR) con sus intervalos de confianza (1C) del 95 %.

De las medidas descritas, la mayor utilidad clínica correspondió al LR, por lo que se hizo énfasis en estos resultados. El LR se define como la probabilidad de dicho resultado en presencia de enfermedad dividida por la probabilidad de dicho resultado en ausencia de enfermedad. Los LR resumen información de la sensibilidad y de la especificidad e indican la capacidad de la prueba para incrementar o disminuir la verosimilitud de un determinado diagnóstico ^34^. Según la interpretación de los LR establecidas por el *Evidence-Based Medicine Working Group*^35^, si el LR es igual a 1, la prueba no tiene utilidad. En la práctica clínica, un LR positivo mayor de 10 o menor de 0,1 es muy útil. La exactitud de la prueba se refiere a la probabilidad general de que un paciente sea correctamente clasificado utilizándola y se determina mediante la proporción de verdaderos positivos y verdaderos negativos entre todos los casos evaluados ^34^.

Para el diseño del estudio, se consideraron los parámetros propuestos por la iniciativa *Standards for Reporting of Diagnostic Accuracy* (STARD) ^36^, la cual pretende mejorar la calidad de los reportes de los estudios de exactitud diagnóstica.

El protocolo fue aprobado por el Instituto de Investigaciones Médicas de la Facultad de Medicina de la Universidad de Antioquia (Acta de aprobación CODI 2010 - N° 580) y por los comités de ética e investigación de ambas instituciones hospitalarias participantes.

## Resultados

En el período del estudio (del 1^o^ de noviembre del 2010 al 1^o^ de febrero del 2014), se incluyeron 46 pacientes con fiebre de origen desconocido, 40 de los cuales cumplieron con el período mínimo de seguimiento (dos fallecieron antes de cumplir los seis meses de seguimiento sin un diagnóstico definitivo de la causa de la fiebre y cuatro se perdieron durante el seguimiento).

Después de una mediana de tiempo de ocho meses (RIC=6-16), el 52,5 % de los 40 pacientes tuvo fiebre de origen desconocido secundaria a una enfermedad reumatológica y el 47,5 % por otras causas. En 11 de los pacientes, se confirmó el diagnóstico de artritis idiopática juvenil sistémica y 29 tuvieron otros diagnósticos y fueron agrupados así: diez (25 %), otras enfermedades reumatológicas; siete (17,5 %), causas infecciosas; dos (5 %), linfohistiocitosis hemofagocítica primaria; dos (5 %), neoplasias malignas o benignas; tres (7,5 %), causas misceláneas, y cinco (12,5 %), fiebre de origen desconocido resuelta sin causa definida (cuadro 1). En el grupo de pacientes con artritis idiopática juvenil sistémica, tres (27 %) tuvieron Ferritina en artritis idiopática juvenil y en otras fiebres de origen desconocido diagnóstico de síndrome de activación macrofágica definido con base en los criterios de la *Pediatric Rheumatology International Trials Organization*^37^. En el grupo de pacientes con fiebre de origen desconocido por otras causas, dos presentaban linfohistiocitosis hemofagocítica primaria confirmada por estudio genético, y otro de estos pacientes con fiebre secundaria a infección por el virus de Epstein Barr tuvo linfohistiocitosis hemofagocítica secundaria demostrada por aspirado de médula ósea.

**Cuadro 1:** Distribución por causas de 40 pacientes remitidos a reumatologia pediátrica por fiebre de origen desconocido

Diagnósticos			n (%)
Causas reumatológicas			21 (52,5)
	Artritis idiopática juvenil sistémica		11 (27,5)
	Enfermedad de Kawasaki		3 (7,5)
	Lupus eritematoso sistémico		2 (5)
	Vasculitis urticariforme		1 (2,5)
	Poliarteritis nodosa		2 (5)
	Síndrome periódico asociado al receptor del TNF (TRAPS)		2 (5)
Otras causas de fiebre de origen desconocido			19 (47,5)
	Causas infecciosas		7 (17,5)
		Fiebre tifoidea	2 (5)
		Infección viral asociada a linfohistiocitosis	1 (2,5)
		Toxoplasmosis	1 (2,5)
		Abscesos intrabdominales en paciente con fibrosis quística	1 (2,5)
		Empiema subdural	1 (2,5)
		Síndrome de infección recurrente anormal en paciente inmunodeficiente	1 (2,5)
Linfohistiocitosis hemofagocítica primaria			2 (5)
	Neoplasias		2 (5)
		Leucemia mieloide	1 (2,5)
		Tumor miofibroblástico inflamatorio	1 (2,5)
	Misceláneas		3 (7,5)
		Fiebre de origen central (diabetes insípida)	1 (2,5)
		Fiebre por antibiótico	1 (2,5)
		Fiebre secundaria a síndrome de hipersensibilidad por carbamazepina	1 (2,5)
	Fiebre de causas no identificadas		5 (12,5)

La edad promedio fue de 7,4 años en los pacientes con artritis idiopática juvenil sistémica y 5,7 años en el grupo de pacientes con fiebre de origen desconocido por otras causas. El 54,5 % de los pacientes con artritis idiopática juvenil sistémica era de sexo masculino (6 pacientes) y el 45,4 % de sexo femenino (5 pacientes); en el grupo de fiebre de origen desconocido por otras causas también predominó el sexo masculino (65,5 %). La duración de la fiebre tuvo una mediana de 40 días (RIC=23-55) en el grupo de artritis idiopática juvenil sistémica, en comparación con una mediana de 26 días (RIC=21-30,5) en el grupo de fiebre de origen desconocido por otras causas.

En comparación con otras causas de fiebre de origen desconocido, los pacientes con artritis idiopática juvenil sistémica presentaron fiebre predominantemente en la noche (p=0,002), y mayor frecuencia de exantema evanescente (p=0,007) y de adenopatías (p=0,02), con una diferencia estadísticamente significativa. No se observaron diferencias con respecto a otras características o manifestaciones clínicas de la fiebre. En el cuadro 2 se presentan las principales características de los exámenes de laboratorio de los pacientes con artritis idiopática juvenil sistémica y aquellos con otras causas de fiebre de origen desconocido. En los primeros, fue más frecuente el aumento de la velocidad de eritrosedimentación por encima de 50 mm/hora y de leucocitosis (para la edad) en comparación con los segundos, con una diferencia estadísticamente significativa (p=0,029).

**Cuadro 2:** Características de laboratorio de los pacientes con artritis idiopática juvenil sistémica y otras causas de fiebre de origen desconocido

Característica	AIJS n=11				Otras causas de FOD§ n=29				
	n (%)	Total n (%)	p^+^	R n=10 (%)	I n=7 (%)	LHH n=2 (%)	N n=2 (%)	M n=3 (%)	NI n=5 (%)
Eritrosedimentación >50 mm/hora	9(81,8)	14(48,3)	0,029	7(70)	3 (42,9)	0	1(50)	0	3(60)
PCR elevada >10 mg/dl	4 (36,4)	5(17,2)	0,227	1(10)	1 (14,3)	0	1(50)	0	2(40)
Proteína C reactiva elevada >1 mg/dl	11 (100)	26 (89,7)	0,548	9(90)	6 (85,7)	2 (100)	2 (100)	3 (100)	4(80)
Anemia (Hb<2 DE según edad y sexo)	9(81,8)	17(58,6)	0,27	6(60)	5(71,4)	0	0	2(66,7)	4(80)
Leucocitosis según edad	7 (63,6)	7(24,1)	0,029	3(30)	2 (28,6)	0	0	0	2(40)
PMNN > 80%	3 (27,3)	4(13,8)	0,369	3(30)	0	0	1 (50)	0	0
AST aumentada	3 (27,3)	11 (37,9)	0,715	2(20)	4 (571)	2 (100)	2 (100)	1 (33,3)	0
ALT aumentada	4 (36,4)	12 (41,4)	1	2(20)	4 (57,1)	2 (100)	2 (100)	2 (66,7)	0
DHL aumentada (según edad)	8(80)	10(41,7)	0,063	1 (12,5)	3(50)	2 (100)	1 (50)	1 (50)	2(50)

AIJS: artritis idiopática juvenil sistémica, FOD: fiebre de origen desconocido §Otras causas de FOD: R: reumatológicas: poliarteritis nodosa ^2^, TRAPS - síndrome febril asociado al receptor del TNF (2), Kawasaki (3), vasculitis urticariforme (1), lupus eritematoso sistémico (2); I: Infecciosas: fiebre tifoidea (2), infección viral asociada a linfohistiocitosis (1), toxoplasmosis (1), abscesos intrabdominales en paciente con fibrosis quística (1), empiema subdural (1), síndrome de infección recurrente anormal en paciente inmunodeficiente (1); LHH: linfohistiocitosis hemofagocítica primaria (2); N: neoplasias: leucemia mieloide (1), tumor miofibroblástico inflamatorio (1); M: misceláneas: fiebre de origen central - diabetes insípida (1), fiebre por antibiótico (1), fiebre secundaria a síndrome de hipersensibilidad por carbamazepina (1); NI: FOD resuelta por causa no identificada (5). PMNN: *polymorphonuclear neutrophil*; AST: aspartato aminotransferasa ALT: alanino aminotransferasa DHL: deshidrogenasa láctica. Valor p *Prueba de ji al cuadrado

+Prueba exacta de Fisher

La mediana de la ferritina total fue más elevada en los pacientes con artritis idiopática juvenil sistémica (3.992 ng/ml; RIC=2.306-9.466 ng/ml) que en aquellos con fiebre de origen desconocido por otras causas (155 ng/ mi; RIC=57-669 ng/ml), con una diferencia estadísticamente significativa (p=0,0027), así como la del nivel de ferritina cinco veces por encima del límite superior para la edad y el sexo, el cual se detectó en 10 de 11 pacientes con artritis idiopática juvenil sistémica (90,91 %) y en 15 de 29 pacientes con fiebre de origen desconocido por otras causas (51,72 %) (p=0,023).

La mediana de la ferritina glucosilada fue de 308,62 ng/ml (RIC=72,4-449) en pacientes con artritis idiopática juvenil sistémica, en comparación con 14,36 ng/ml (RIC=7,7-30,7) en aquellos con fiebre de origen desconocido por otras causas y la diferencia fue estadísticamente significativa (p=0,0005). Se observó un mayor porcentaje de pacientes con ferritina glucosilada menor de 20 % de la total en el grupo de pacientes con otras causas de fiebre de origen desconocido (96,5 %), en comparación con aquellos con artritis idiopática juvenil sistémica (81,8 %), pero la diferencia no fue estadísticamente significativa (p=0,178) (cuadro 3).

**Cuadro 3:** Comparación de los resultados de la ferritina total, la ferritina glucosilada y el valor de ferritina cinco veces por encima del normal (según sexo y edad) entre pacientes con artritis idiopática juvenil sistémica y otras causas de fiebre de origen desconocido

Prueba diagnóstica	AIJS (n=11)	Otras causas de FOD (n=29)	p
Ferritina sérica total en ng/ml, mediana (RIC)	3.992 (2.306-9.466)	155 ng/ml (57-669)	0,0027^+^
Ferritina mayor a 5 veces lo normal, n (%)	90,91	51,72	0,023*
Ferritina glucosilada en ng/ml, mediana v(RIC)	308,62 (72,4-449)	14,36 (7,7-30,7)	0,0005^+^
Ferritina glucosilada menor de 20 % de la total, n (%)	81,82	96,55	0,178*

AIJS: artritis idiopática juvenil sistémica, FOD: fiebre de origen desconocido

Valor p: + Mann-Whitney; * Prueba exacta de Fisher

En los cuadros 4 y 5 se presenta la información sobre las medidas de utilidad de las pruebas diagnósticas. Al analizar la ferritina total con un valor 5 veces por encima del normal (según sexo y edad) (cuadro 4), se encontró una sensibilidad del 91 % (IC_950/_ 58,72 %-99,77 %) y una especificidad del 48 % (IC_95%_ 29,45 %-67,47 %), un LR positivo de 1,76 (IC_95%_ 1,18-2,62), un LR negativo de 0,19 (IC_95%_ 0,03-1,27), en tanto que la exactitud de la prueba fue del 60 %. Según la interpretación de los LR ya mencionada (*Evidence-Based Medicine Working Group*) ^35^, el LR positivo de 1,76 cambia la probabilidad diagnóstica en un grado insignificante, en tanto que un LR negativo de 0,19 (IC_95%_ 0,03-1,27) indica un cambio moderado de la probabilidad diagnóstica, lo que, asociado con una sensibilidad del 91 %, indica que es un método importante para la tamización de la enfermedad.

**Cuadro 4:** Valor de ferritina 5 veces por encima del normal (según sexo y edad) para el diagnóstico de artritis idiopática juvenil sistémica Vs. fiebre de origen desconocido por otras causas

Ferritina 5 veces por encima del valor normal*	Pacientes con AIJS	Otras causas de FOD	Total
Prueba positiva	10 VP (a)	15 FP (b)	25
Prueba negativa	1 FN (c)	14 VN (d)	15
Total	11 (a+c)	29 (b+d)	40

AIJS: artritis idiopática juvenil sistémica, FOD: fiebre de origen desconocido

* Valores de referencia de ferritina según edad y sexo: para niños de ambos sexos de 1 a 5 años, hasta 24 ng/ml, y de 6 a 10 años, hasta 55 ng/ml; de los 11 a los 16 años, para el sexo masculino hasta 70 ng/ml y para el femenino hasta 40 ng/ml ^33^.

(a) : Verdaderos positivos (VP): AIJS con prueba positiva

(b) : Falsos positivos (FP): otras causas de FOD con prueba positiva

(c) : Falsos negativos (FN): AIJS con prueba negativa

(d) : Verdaderos negativos (VN): otras causas de FOD con prueba negativa a+c: casos con patrón de referencia positivo (AIJS)

b+d: casos con patrón de referencia negativo (otras causas de FOD) a+b: casos con la prueba diagnóstica positiva (ferritina 5 veces por encima del valor normal)

c+d: casos con la prueba diagnóstica negativa (ferritina 5 veces por encima del valor normal)

Sensibilidad (S) = 91 % (IC95% 58,72-99,77 %)

Especificidad (E) = 48% (IC95% 29,45-67,47 %)

Valor predictivo positivo (VPP) = 40,0 % (IC95% 30,92-49,82 %)

Valor predictivo negativo (VPN) = 93,3% (1095% 67.54-98,95 %)

Proporción de falsos positivos (FP)= 52% (1095% 34,4-68,6 %)

Proporción de falsos negativos (FN)= 9% (1095% 1,6-37,7 %)

Exactitud=60 % (1095% 43,3-75,1 %)

Cociente de probabilidad positivo (LR+) = 1,76 (1095% 1,18-2,62)

Cociente de probabilidad negativo (LR-) = 0,19 (1095% 0,03-1,27)

Probabilidad pre-prueba (prevalencia) = 27,5 % (1095% 14,60-43,89 %)

**Cuadro 5:** Prueba combinada de ferritina 5 veces por encima del valor normal (según sexo y edad) y ferritina glucosilada menor de o igual al 20 % de la total para el diagnóstico de artritis idiopática juvenil sistémica Vs. fiebre de origen desconocido por otras causas

Ferritina 5 veces por encima del valor normal*	Pacientes con AIJS	Otras causas de FOD	Total
Prueba positiva	9 VP (a)	15 FP (b)	24
Prueba negativa	2 FN (c)	14 VN (d)	16
Total	11 (a+c)	29 (b+d)	40

AIJS: artritis idiopática juvenil sistémica, FOD: fiebre de origen desconocido

*Valores de referencia de ferritina según edad y sexo: para niños de ambos sexos de 1 a 5 años, hasta 24 ng/ml, y de 6 a 10 años, hasta 55 ng/ml; de los 11 a los 16 años, para el sexo masculino hasta 70 ng/ml y para el femenino hasta 40 ng/ml ^33^.

(a) : Verdaderos positivos (VP): AIJS con prueba positiva

(b) : Falsos positivos (FP): otras causas de FOD con prueba positiva

(c) : Falsos negativos (FN): AIJS con prueba negativa

(d) : Verdaderos negativos (VN): otras causas de FOD con prueba negativa a+c: casos con patrón de referencia positivo (AIJS)

b+d: casos con patrón de referencia negativo (otras causas de FOD) a+b: casos con la prueba diagnóstica positiva (ferritina 5 veces por encima del valor normal y ferritina glucosilada menor o igual al 20 % de la total) c+d: casos con la prueba diagnóstica negativa (ferritina 5 veces por encima del valor normal y ferritina glucosilada menor o igual al 20 % de la total)

Sensibilidad (S)=81,8 % (IC95% 52,3-94,9 %)

Especificidad (E)=48,3 % (1095% 31,4-65,6 %)

Valor predictivo positivo (VPP) = 37,5 % (1095% 21,2-57,3 %)

Valor predictivo negativo (VPN) = 87,5 % (1095% 64,0-96,5 %)

Proporción de falsos positivos (FP)= 51,7 (1095% 34,4-68,6 %)

Proporción de falsos negativos (FN)= 51,7 (1095% 34,4-68,6 %)

Exactitud=57,5 % (1095% 42,2-71,5 %)

Cociente de probabilidades positivo (LR+)=1,58 (1095% 1,01-2,48)

Cociente de probabilidades negativo (LR-)=0,38 (1095% 0,10-1,41)

Probabilidad pre-prueba (prevalencia)=27,5 %

En cuanto a la ferritina glucosilada menor de o igual al 20 % de la ferritina total, se encontró una sensibilidad del 91 % (IC_95%_ 62,3-98,4 %) pero una especificidad del 3,4 % (IC_950%_ 0,6-17,2 %); la exactitud de la prueba fue del 27,5 % (IC_95%_ 16,1-43,8 %) y el LR positivo fue de 0,94 (IC_95%_ 0,77-1,15), muy cercano al 1, lo que altera la probabilidad diagnóstica en un grado insignificante.

En el cuadro 5, se presenta la información sobre las medidas de utilidad de las pruebas combinadas (ferritina total mayor de cinco veces el valor normal y el porcentaje de la ferritina glucosilada menor de o igual al 20 % de la total). La prueba combinada tuvo una sensibilidad del 81,8 % (IC_95%_ 52,3- 94,9 %) y una especificidad del 48,3 % (IC_950%_ 31,4-65,6 %); la exactitud fue del 57,5 % (IC_95%_ 42,2-71,5 %) y el LR positivo de 1,58 (1C 1,01-2,48), lo que cambia la probabilidad diagnóstica en un grado insignificante, en tanto que el LR negativo fue de 0,38 (IC_95%_ 0,10-1,41), lo que genera un cambio pequeño en dicha probabilidad.

## Discusión

El término artritis idiopática juvenil propuesto por la ILAR agrupa según categorías siete enfermedades diferentes, incluida la artritis idiopática juvenil sistémica, que representa entre el 4 y el 17 % del total de pacientes con artritis idiopática juvenil ^3^. Esta enfermedad frecuentemente debuta como una fiebre de origen desconocido, por lo que el manejo clínico es complejo dado que, por definición, la artritis idiopática juvenil sistémica es un diagnóstico de exclusión. Con el propósito de determinar la etiología de la fiebre de origen desconocido en población pediátrica general, Chow, *et al*., hicieron una revisión sistemática de la literatura y encontraron que las enfermedades reumáticas constituyeron el 9 % de todas las causas de fiebre de origen desconocido, siendo la artritis idiopática juvenil sistémica la más común en esta categoría ^31^, con un 5,5 %. Teniendo en cuenta que en esta serie se incluyeron solamente pacientes remitidos a reumatología pediátrica Ferritina en artritis idiopática juvenil y en otras fiebres de origen desconocido por fiebre de origen desconocido, la frecuencia de enfermedades reumáticas fue mucho mayor (52,5 %) y la artritis idiopática juvenil sistémica representó el 27,5 % del total de las causas.

Desde el punto de vista clínico y paraclínico, en comparación con las otras causas de fiebre de origen desconocido y las diferencias estadísticamente significativas, los pacientes con artritis idiopática juvenil sistémica tuvieron mayor frecuencia de fiebre de predominio nocturno, exantema evanescente, adenopatías, aumento de la velocidad de eritrosedimentación (mayor de 50 mm/hora) y leucocitosis (cuadro 2); sin embargo, otras manifestaciones como la artritis, u otros marcadores como la proteína C reactiva elevada (mayor de 10 mg/dl), que refleja el intenso proceso inflamatorio característico de la enfermedad, no alcanzaron una diferencia estadísticamente significativa con respecto a las otras causas de fiebre de origen desconocido (cuadro 2). Los hallazgos descritos evidencian la dificultad para diferenciar la artritis idiopática juvenil sistémica de otras causas de fiebre de origen desconocido con base en los criterios clínicos y de laboratorio convencionales, dificultad que se incrementa por el hecho de que el tratamiento de la dicha artritis con esteroides y otros medicamentos inmunosupresores puede empeorar algunas de las otras enfermedades, como infecciones o neoplasias.

La ferritina plasmática es una proteína cuya producción aumenta durante los estados inflamatorios (16-18). El incremento es extremadamente alto en pacientes con enfermedad de Still del adulto y, puesto que es un recurso de laboratorio accesible, varios autores lo han propuesto como marcador de actividad para esta enfermedad ^10^^,^^20^^,^^21^. En el 2001, se propuso que, además de la hiperferritinemia, el hallazgo de una ferritina glucosilada menor de o igual al 20 % de la total podría mejorar el rendimiento diagnóstico de la prueba para esta enfermedad ^10^ y, en el 2002, se incluyó como uno de los principales criterios en una propuesta de clasificación ^25^, la cual fue validada en el 2018 ^26^.

El aumento de la ferritina se ha informado en algunas series descriptivas de pacientes con artritis idiopática juvenil sistémica ^5^^,^^27^. Behrens, *et al.*, encontraron, en una cohorte de 136 pacientes con artritis idiopática juvenil sistémica, que la ferritina era mayor de 500 ng/ml en 46 de 66 pacientes (70 %) ^5^. En un estudio descriptivo en Colombia, Ramírez, *et al.*, encontraron que, en el 78 % de 15 pacientes con artritis idiopática juvenil sistémica, los niveles de ferritina estaban cinco veces por encima del valor normal para la edad y el sexo ^27^. En la presente serie, este porcentaje fue mayor (90,91 %) (cuadro 3), hallazgo que podría estar relacionado con una mayor frecuencia del síndrome de activación macrofágica (27 %) en comparación con otras series en las cuales se ha registrado en cerca del 10 % de los pacientes ^2^. Sobieska, *et al.*, evaluaron las proteínas de fase aguda en 10 pacientes con artritis idiopática juvenil sistémica en comparación con 36 muestras de 27 pacientes con enfermedad de Still del adulto (21 muestras en fase activa y 15 en remisión), y encontraron valores de ferritina total muy elevados en aquellos con la enfermedad activa (promedio de 2.385 ng/ml±1.517), en tanto que en los enfermos con artritis idiopática juvenil sistémica artritis idiopática juvenil sistémica y con la enfermedad en remisión, los valores mostraron un incremento leve (218 ng/ml±101 y 240 ng/ml±106, respectivamente) ^28^.

Hasta donde fue posible establecerlo, no hay en la literatura estudios de pruebas diagnósticas sobre la utilidad de la ferritina con un valor cinco veces por encima del normal ni de la ferritina glucosilada en la artritis idiopática juvenil sistémica. Por ello, se llevó a cabo este estudio considerando como patrón diagnóstico de referencia el cumplimiento de los criterios de la ILAR o del pediatra reumatólogo durante un seguimiento de mínimo 12 meses, como se determinó en la metodología.

En comparación con el grupo de pacientes con fiebre de origen desconocido por otras causas, en aquellos con artritis idiopática juvenil sistémica la ferritina total tuvo un aumento estadísticamente significativo (cuadro 3). En este estudio, el valor de la ferritina cinco veces por encima del normal (según sexo y edad) tuvo una sensibilidad del 91 % (IC_95%_ 58,72-99,77 %) y una especificidad del 48 % (IC_95%_ 29,45-67,47 %) para el diagnóstico de la artritis idiopática juvenil sistémica (cuadro 4). En un estudio sobre el valor diagnóstico de los niveles de ferritina total y ferritina glucosilada para la enfermedad de Still del adulto, Fautrel, *et al.*, compararon dichos niveles en 49 pacientes y 120 controles con enfermedades infecciosas, neoplásicas y otras enfermedades sistémicas ^10^.

En comparación con el presente estudio, el valor de ferritina cinco veces por encima del normal tuvo una sensibilidad del 40,8 % y una especificidad del 80 %, lo cual podría explicarse porque, en el estudio de Fautrel, *et al.*, en el 20,4 % de los pacientes la enfermedad de Still del adulto estaba inactiva en el momento de la inclusión ^10^, en tanto que, en el presente estudio, la toma de muestra para la ferritina total y la glucosilada se hizo en el momento de la evaluación inicial en el servicio de reumatología pediátrica, cuando todos los pacientes tenían fiebre y otras manifestaciones de enfermedad activa.

En relación con la menor especificidad, en el presente estudio, se explicaría porque los pacientes con linfohistiocitosis hemofagocítica primaria tuvieron valores similares (un poco más elevados) de ferritina total que aquellos con artritis idiopática juvenil sistémica. La linfohistiocitosis hemofagocítica es una enfermedad infrecuente caracterizada por fiebre, hepatoesplenomegalia, pancitopenia y niveles muy elevados de ferritina que se relacionan con la proliferación y activación de macrófagos benignos por hemofagocitosis en la médula ósea ^38^. Existen dos formas: la primaria, que es una enfermedad autosómica recesiva, y la secundaria, asociada con infección, neoplasia y enfermedades autoinmunitarias, incluida la artritis idiopática juvenil sistémica, en las cuales se denomina síndrome de activación macrofágica. En varios estudios se ha demostrado que la hiperferritinemia y la disminución de la ferritina glucosilada son hallazgos característicos de la linfohistiocitosis hemofagocítica primaria y la secundaria ^39^^-^^41^.

La ferritina glucosilada menor de o igual al 20 % del valor total de la ferritina tuvo una sensibilidad del 91 % (IC_95%_ 62,3-98,4 %), pero una especificidad muy baja, del 3,4 % (IC_95%_ 0,6-17,2 %), para el diagnóstico de artritis idiopática juvenil sistémica. Este dato contrasta con los hallazgos del estudio de Fautrel, *et al.*, en el cual se registró una sensibilidad adecuada, del 79,5 %, y una especificidad del 66,4 % ^10^. No hay una explicación clara para este hallazgo y, aunque comparar cualquiera de los resultados de estos dos estudios entraña la dificultad de que se trata de metodologías y poblaciones diferentes (niños y adultos), la mayoría de autores consideran que la enfermedad de Still del adulto y la artritis idiopática juvenil sistémica son la misma pero en edades opuestas ^42^^-^^44^. En este sentido, la comparación de los resultados del presente estudio se dificulta, pues no hay otros publicados sobre el valor diagnóstico de la ferritina total y la glucosilada en niños. Además de la ferritina total, Sobieska, *et al.*^28^, evaluaron otras Ferritina en artritis idiopática juvenil y en otras fiebres de origen desconocido proteínas como la glucoproteína ácida alfa 1 y la antiquimotripsina alfa 1, y registraron su aumento en los casos de enfermedad de Still del adulto y su disminución en aquellos con artritis idiopática juvenil sistémica (p<0,01). Estos autores plantean que, dado que la diferencia en los perfiles de glucosilación de estas proteínas indicaría mecanismos patogénicos disímiles en las dos enfermedades, es cuestionable que pueden considerarse equivalentes ^28^.

El valor de la ferritina total cinco veces por encima del normal y el de la glucosilada menor de o igual al 20 % de la total (cuadro 5), tuvieron en este estudio una sensibilidad del 81,8 % (IC_95%_ 52,3-94,9 %) y una especificidad del 48,3 % (IC_95%_ 31,4-65,6 %), en comparación con 43,2 y 92,9 %, respectivamente, reportados en el estudio de Fautrel, *et al.*^10^. Al analizar las medidas de utilidad de las pruebas diagnósticas, la más útil es la del LR, ya que resume la información de la sensibilidad y la especificidad, e indica la capacidad de la prueba para incrementar o disminuir la verosimilitud de un determinado diagnóstico ^34^. Para la prueba combinada, un LR positivo de 1,58 (IC_95%_ 1,01-2,48) cambia la probabilidad diagnóstica en un grado insignificante, en tanto que un LR negativo de 0,38 (IC_95%_ 0,10-1,41) genera un cambio pequeño en la probabilidad diagnóstica ^35^. Los resultados fueron mejores para el valor de ferritina total cinco veces por encima del normal (según sexo y edad), ya que el LR positivo de 1,76 (IC_95%_ 1,18-2,62) cambia la probabilidad diagnóstica en un grado insignificante, pero un LR negativo de 0,19 (IC_95%_ 0,03-1,27) indica un cambio moderado de la probabilidad diagnóstica, lo que conjuntamente con una sensibilidad del 91 % indica que es un método importante para la tamización de la enfermedad en pacientes pediátricos con fiebre de origen desconocido, quienes requerirían la evaluación por reumatología y hematología pediátrica. Desde el punto de vista práctico, este resultado es relevante, ya que, a diferencia de la ferritina glucosilada, la ferritina sérica es un recurso de laboratorio accesible.

En cuanto a las principales limitaciones del estudio, una fue la realización de las pruebas en un solo momento de la evolución de la enfermedad; aunque la toma de la muestra se hizo en la evaluación inicial por reumatología pediátrica, al comparar entre pacientes no es posible asegurar que todos estuvieran en el mismo momento de evolución clínica y, por lo tanto, los marcadores de inflamación pudieron variar. La otra limitación fue el no haber contado en la práctica clínica con la prueba de la ferritina glucosilada, pues esta se estandarizó específicamente para este estudio; sin embargo, dados los hallazgos descritos y la ausencia de otros datos en población pediátrica, se requieren nuevos estudios para entender los mecanismos que inducen la producción excesiva de ferritina y constatar si en la artritis idiopática juvenil sistémica difieren de los resultados encontrados en la enfermedad de Still del adulto.

En pacientes evaluados por reumatología pediátrica debido a fiebre de origen desconocido, los valores de la ferritina total cinco veces por encima de los normales (según sexo y edad) demostraron su utilidad para la tamización de la artritis idiopática juvenil sistémica y un mejor desempeño que la combinación de esta misma prueba con la ferritina glucosilada menor o igual al 20 % del valor total.

## Archivos suplementarios

**Anexo 1.** Metodología para el procesamiento de la ferritina glucosilada

El suero del paciente se tomó en tubo sin anticuagulante. Después de retraer el coágulo, se llevó a la centrífuga Hettich EBA -20 a 4.000 revoluciones por minuto (r.p.m.) por 10 minutos y, posteriormente, se congeló a -80 °C.

Se utilizaron los reactivos Sepharosa 4B X 500 mL SIGMA Ref 4B200 y Concanavalina A (Con A) X 250 mg SIGMA Ref C-7275. Dichos reactivos y la muestra del paciente se dejaron a temperatura ambiente dos horas antes de ser utilizados.

**Procedimiento:**

1. Se utilizaron tubos de 50 mi de tipo Falcon BD (352098 Falcon), a los cuales se les introdujo una cámara con una membrana para filtración hecha a base de lana de vidrio (PANREAC por 100 g) con un peso de 1,5 gramo, quedando con un diámetro aproximado de 2 x 2 cm. Posteriormente, estos tubos se esterilizaron en formaldehido durante seis horas a 55 °C.

2. Se resuspendió la Con A en 10 mi de agua destilada para obtener 25 mg/ml, que debe llevarse a una potencia menor de 64 pg/ml. La Sepharosa utilizada tiene una capacidad de saturación de ≥30 mg/ml.

3. Se humedeció la membrana con 3 mi de agua destilada de forma homogénea, teniendo la precaución de añadir a un mi de agua. Después se llevó a la centrífuga Thermo Scientific Sorvall RT3 refrigerada y se realizó un giro hasta llegar a 3.000 r.p.m. para remover el agua utilizada.

4. Se montaron seis tubos debidamente marcados por paciente: los tres primeros como Sepharosa 4B y los tres últimos como Con A más Sepharosa 4B. A todos los tubos se les adicionaron 100 pl de suero del paciente y 100 pl más de cada reactivo, según correspondiera. Se dejaron en el agitador de Mazzini universal, modelo OS-20, por dos horas a 150 r.p.m.

100 μl Sepharosa 4B +100 μl suero/tubo 100 μl ConA+ 100μl Sepharosa 4B+100 μl suero/tubo

5. Se llevaron los tubos a la centrifuga refrigerada por 10 minutos a 4.000 r.p.m.

6. Se separaron 150 μl del sobrenadante en tubos de Eppendorf de 1,5 mi Gentech debidamente marcados. Posteriormente, se hizo el imnunoanálisis quimioluminiscente de partículas para determinar la ferritina. El estuche utilizado fue el 7K59 ARCHITECT.

Los valores obtenidos se utilizaron en la siguiente fórmula para determinar el porcentaje de ferritina glucosilada (% FG).

La ferritina glucosilada se determinó incubando el suero con Con A-sefarosa y luego se mezcla durante dos horas a temperatura ambiente. Se centrifugó a 3.000 r.p.m. por 15 minutos para remover la ferritina glucosilada del total. La ferritina no glucosilada no se unió a la Con A y se recuperó en el sobrenadante para ser cuantificada nuevamente por inmunoensayo. De forma similar, el suero se incubó con sefarosa 4B y la ferritina medida en el sobrenadante correspondió a la ferritina sérica total. La ferritina glucosilada corresponde a la diferencia entre la ferritina total y la ferritina no ligada, y se expresa en términos de porcentaje de la ferritina total.

Técnica basada en: Worwood M, Cragg SJ, Wagstaff M, Jacobs A. Binding of human serum ferritin to concanavalin A. Clin Sci. 1979;56:83-7.

## References

[1] Lee JJY, Schneider R (2018). Systemic juvenile idiopathic arthritis. Pediatr Clin North Am.

[2] Minoia F, Davì S, Horne A, Demirkaya E, Bovis F, Li C (2014). Clinical features, treatment, and outcome of macrophage activation syndrome complicating systemic juvenile idiopathic arthritis: A multinational, multicenter study of 362 patients. Arthritis Rheumatol.

[3] Petty RE, Southwood TR, Manners P, Baum J, Glass DN, Goldenberg J (2004). International League of Associations for Rheumatology Classification of Juvenile Idiopathic Arthritis. J Rheumatol.

[4] Martini A (2012). It is time to rethink juvenile idiopathic arthritis classification and nomenclature. Ann Rheum Dis.

[5] Behrens EM, Beukelman T, Gallo L, Spangler J, Rosenkranz M, Arkachaisri T (2008). Evaluation of the presentation of systemic onset juvenile rheumatoid arthritis: Data from the Pennsylvania Systemic Onset Juvenile Arthritis Registry (PASOJAR). J Rheumatol.

[6] Hinze CH, Holzinger D, Lainka E, Haas JP, Speth F, Kallinich T (2018). Practice and consensus-based strategies in diagnosing and managing systemic juvenile idiopathic arthritis in Germany. Pediatr Rheumatol.

[7] Martini A, Ravelli A, Avcin T, Beresford MW, Burgos-Vargas R, Cuttica R (2019). Toward new classification criteria for juvenile idiopathic arthritis: First steps, pediatric rheumatology international trials organization international consensus. J Rheumatol.

[8] Andreola B, Bressan S, Callegaro S, Liverani A, Plebani M, Da Dalt L (2007). Procalcitonin and C-reactive protein as diagnostic markers of severe bacterial infections in febrile infants and children in the emergency department. Pediatr Infect Dis J.

[9] Foell D, Wittkowski H, Hammerschmidt I, Wulffraat N, Schmeling H, Frosch M (2004). Monitoring neutrophil activation in juvenile rheumatoid arthritis by S100A12 serum concentrations. Arthritis Rheum.

[10] Fautrel B, Le Moël G, Saint-Marcoux B, Taupin P, Vignes S, Rozenberg S (2001). Diagnostic value of ferritin and glycosylated ferritin in adult onset Still’s disease. J Rheumatol.

[11] Kernan KF, Carcillo JA (2017). Hyperferritinemia and inflammation. Int Immunol.

[12] Ghosh S, Hevi S, Chuck SL (2004). Regulated secretion of glycosylated human ferritin from hepatocytes. Blood.

[13] Koorts AM, Viljoen M (2007). Ferritin and ferritin isoforms I: Structure-function relationships, synthesis, degradation and secretion. Arch Physiol Biochem.

[14] Cazzola M (2005). Role of ferritin and ferroportin genes in unexplained hyperferritinaemia. Best Pract Res Clin Haematol.

[15] Yamanishi H, Kimura S, Hata N, lyama S, Kanakura Y, Iwatani Y (2007). Evaluation of a model of latent pathologic factors in relation to serum ferritin elevation. Clin Biochem.

[16] García PC, Longhi F, Branco RG, Piva JP, Lacks D, Tasker RC (2007). Ferritin levels in children with severe sepsis and septic shock. Acta Paediatr Int J Paediatr.

[17] Bennett TD, Hayward KN, Farris RWD, Ringold S, Wallace CA, Brogan T V (2011). Very high serum ferritin levels are associated with increased mortality and critical care in pediatric patients. Pediatr Crit Care Med.

[18] D’Souza B, Sinha S, Manjrekar P, D’Souza V (2013). Hyperferritinemia in pulmonary tuberculosis. Indian J Clin Biochem.

[19] Fautrel B (2008). Adult-onset Still disease. Best Pract Res Clin Rheumatol.

[20] Panqueva U, Ramírez LA, Restrepo JF, Rondón F, Mora S, Valle R (2009). Enfermedad de Still del adulto: estudio de cohorte. Rev Colomb Reumatol.

[21] Vignes S, Le Moël G, Fautrel B, Wechsler B, Godeau P, Piette JC (2000). Percentage of glycosylated serum ferritin remains low throughout the course of adult onset Still’s disease. Ann Rheum Dis.

[22] Bilgin E, Hayran M, Erden A, Armağan B, Sari A, Kiliç L (2019). Correction to: Proposal for a simple algorithm to differentiate adult-onset Still’s disease with other fever of unknown origin causes: A longitudinal prospective study. Clin Rheumatol.

[23] Carreño AM, Carvallo A, Trejo C, Ballestero F, Martínez C (2009). Enfermedad de Still del adulto: una gran simuladora. Experiencia clínica basada en 20 casos. Rev Med Chil.

[24] Eloseily EMA, Minoia F, Crayne CB, Beukelman T, Ravelli A, Cron RQ (2019). Ferritin to erythrocyte sedimentation rate ratio: Simple measure to identify macrophage activation syndrome in systemic juvenile idiopathic arthritis. ACR Open Rheumatol.

[25] Fautrel B, Zing E, Golmard JL, Le Moei G, Bissery A, Rioux C (2002). Proposal for a new set of classification criteria for adult-onset still disease. Medicine (Baltimore).

[26] Lebrun D, Mestrallet S, Dehoux M, Golmard JL, Granger B, Georgin-Lavialle S (2018). Validation of the Fautrel classification criteria for adult-onset Still’s disease. Semin Arthritis Rheum.

[27] Ramirez LA, Prada MC, Alarcón P, Díaz A, Eraso R (2004). Utilidad de la ferritina sérica en el diagnóstico de pacientes con artritis reumatoide juvenil de tipo sistémico. Rev Colomb Reumatol.

[28] Sobieska M, Fassbender K, Aeschlimann A, Bourgeois P, Mackiewicz S, Müller W (1998). Still’s disease in children and adults : A distinct pattern of acute-phase proteins. Clin Rheumatol.

[29] Marshall GS (2014). Prolonged and recurrent fevers in children. J Infect.

[30] Long SS (2005). Distinguishing among prolonged, recurrent, and periodic fever syndromes: Approach of a pediatric infectious diseases subspecialist. Pediatr Clin North Am.

[31] Chow A, Robinson JL (2011). Fever of unknown origin in children: A systematic review. World J Pediatr.

[32] Worwood M, Cragg SJ, Wagstaff M, Jacobs A (1979). Binding of human serum ferritin to concanavalin A. Clin Sci (Lond).

[33] Brugnara C (2009). Reference values in infancy and childhood. In: Orkin S. Nathan and Oski’s hematology of infancy and childhood.

[34] Ochoa-Sangrador C (2015). AEPap ed. Curso de Actualización Pediatría 2015.

[35] Jaeschke R, Guyatt G, Sackett D (1994). Users’ guides to the medical literature. III. How to use an article about a diagnostic test. B. What are the results and will they help me in caring for my patients? The Evidence-Based Medicine Working Group. JAMA.

[36] Bossuyt PM, Reitsma JB, Bruns DE, Gatsonis CA, Glasziou PP, Irwig LM (2003). The STARD statement for reporting studies of diagnostic accuracy: Explanation and elaboration. Croat Med J.

[37] Ravelli A, Minoia F, Davi S, Horne AC, Bovis F, Pistorio A (2016). Classification criteria for macrophage activation syndrome complicating systemic juvenile idiopathic arthritis: A European league against Rheumatism/American college of Rheumatology/Paediatric rheumatology international trials organisation collaborative initiative. Ann Rheum Dis.

[38] Henderson LA, Cron RQ (2020). Macrophage activation syndrome and secondary hemophagocytic lymphohistiocytosis in childhood inflammatory disorders: Diagnosis and management. Pediatr Drugs.

[39] Wang Z, Wang Y, Wang J, Feng C, Tian L, Wu L (2009). Early diagnostic value of low percentage of glycosylated ferritin in secondary hemophagocytic lymphohistiocytosis. Int J Hematol.

[40] Fardet L, Coppo P, Kettaneh A, Dehoux M, Cabane J, Lambotte O (2008). Low glycosylated ferritin, a good marker for the diagnosis of hemophagocytic syndrome. Arthritis Rheum.

[41] Kwok JSS, Wong PCH, Luk MC, Chan MHM (2012). Use of glycosylated ferritin assay to aid the diagnosis of adult-onset Still’s disease: A local laboratory experience in Hong Kong. Rheumatol Int.

[42] Pay S, Türkçapar N, Kalyoncu M, Şimşek I, Beyan E, Ertenli I (2006). A multicenter study of patients with adult-onset Still’s disease compared with systemic juvenile idiopathic arthritis. Clin Rheumatol.

[43] Jamilloux Y, Gerfaud-Valentin M, Martinon F, Belot A, Henry T, Sève P. (2014). Pathogenesis of adult- onset Still’s disease: New insights from the juvenile counterpart. Immunol Res.

[44] Inoue N, Shimizu M, Tsunoda S, Kawano M, Matsumura M, Yachie A (2016). Cytokine profile in adult-onset Still’s disease: Comparison with systemic juvenile idiopathic arthritis. Clin Immunol.

